# Poverty Reduction and Family Functioning: Results from an Experimental Study in Sub-Saharan Africa

**DOI:** 10.1007/s10826-024-02920-0

**Published:** 2024-10-02

**Authors:** Leyla Karimli, Josephine Nabayinda, Portia B. Nartey, Fred M. Ssewamala

**Affiliations:** 1 Los Angeles (UCLA), Luskin School of Public Affairs, University of California, 337 Charles E Young Dr E, Los Angeles, CA 90095, USA; 2 International Center for Child Health and Development (ICHAD), University of Washington, Saint Louis, Brown School of Social Work, 1 Brookings Dr, St. Louis, MO 63130, USA

**Keywords:** Family functioning in families affected by HIV/AIDS, Asset-based poverty-reductionintervention, Randomized controlled trial, Sub-Saharan Africa, Family supportfor HIV/AIDS orphans in Uganda

## Abstract

The study tests the effect of poverty-reduction intervention on family functioning reported by AIDS-orphaned children in extended families in Southern Uganda by asking two questions: (1) based on children’s reports, how does poverty reduction intervention affect family functioning? and (2) to what extent do these effects vary by a child’s gender and orphanhood status? Informed by the social causation theory, family stress model, and asset theory, the study aims to address the existing knowledge gap on effects of poverty reduction interventions on family functioning in low-income families caring for AIDS orphaned children in Uganda. We ran multilevel regression models using longitudinal data collected in a cluster-randomized controlled trial from N = 1410 children (n = 621 boys and n = 789 girls) recruited from 48 rural primary schools in Uganda. Survey data was collected every 12 months over the course of 5 years. The average age of children at enrollment was 13 years. We found significant positive effects of the intervention on family cohesion, family communication, and child-caregiver relationship. Effects vary by child’s gender and orphanhood category. Intervention improves family communication for boys, while improving family cohesion and quality of child-caregiver relationship for girls. Single maternal orphans reported improved family communication, while single paternal orphans reported improved child-caregiver relationship. Poverty reduction interventions are important to improve family functioning for low-income families. Variations by child’s gender and orphanhood status have not been reported in previous studies, and our findings underscore the importance of continued research in this area.

## Background

### Poverty and Family Functioning in the Context of Sub-Saharan Africa

Ending poverty in all its forms is one of the key goals of the UN’s 2030 Sustainable Development Agenda, yet extreme poverty remains prevalent in sub-Saharan Africa (SSA), where over 490 million people live below the poverty line, defined as living on less than USD 2 a day (UNCTAD, 2021). SSA also has the highest number of families affected by HIV/AIDS (Disassa & Lamessa, 2021), and, of the 17.8 million children orphaned by HIV/AIDS worldwide, 15.1 million reside in sub-Saharan Africa (Bryant & Beard, 2016). HIV/AIDS combined with poverty constitutes a significant risk factor for socio-economic and emotional well-being of families (Ashaba et al., 2019; Kalomo & Besthorn, 2018). Children who have lost one or both parents to HIV/AIDS often lack family support, placing them at higher risk of adverse experiences, including stigma, depression, anxiety, hopelessness, poor peer relations, and poor educational outcomes, such as reduced attendance and attainment (Ashaba et al., 2019; Cluver et al., 2007; Raymond & Zolnikov, 2018). To cope with the stress associated with parents living with a highly stigmatized chronic disease (HIV) and/or loss of a parent, family disruption, and poverty, children affected by or orphaned due to AIDS rely on extended families—also referred to as family support networks—as a safety net (Heymann et al., 2007; Karimli et al., 2012; Motha, 2018; Nabunya et al., 2019). However, resource constraints and the high prevalence of HIV/AIDS pose significant challenges for many extended families in integrating children orphaned by AIDS (Shava et al., 2016). These children in extended family households frequently face significant resource shortages, often going to bed without a meal and dropping out of school to care for their siblings (Ntuli et al., 2020). Caring for orphaned children places additional demands on caregivers, while the lack of parental care increases the children’s vulnerability to poor mental health outcomes (Disassa & Lamessa, 2021). This situation further strains family functioning—defined as the ability of the family to achieve well-being, adapt to changing circumstances, and balance the needs of individual family members with those of the whole family (Keitner et al., 2009).

The effects of poverty on family functioning are complex, involving economic stress, limited access to resources, and psychological strain. Scarcity of resources often leads to stressful life events and demographic disadvantages, thereby jeopardizing family cohesion (Booysen et al., 2021). Empirical evidence suggests that higher socioeconomic status correlates with stronger family functioning (Botha et al., 2018), while lower income levels are associated with diminished family functioning and an increased reliance on social networks for managing economic stressors (Denny et al., 2014). Additionally, economic hardships can heighten parental stress, leading to disruptions in parenting practices (Conger et al., 2010; Masarik & Conger, 2017). Longitudinal studies consistently show a significant link between economic hardships and reduced family functioning, characterized by increased parental stress and depression, relationship conflicts, reduced parental engagement, and harsh parenting practices. These conditions, in turn, contribute to deteriorating child mental health and an increased likelihood of externalizing behaviors in children (Kavanaugh et al., 2018; Neppl et al., 2016; Simons et al., 2016).

### Poverty Reduction Intervention: Overview and Impact on Family Functioning

This study examines a family-based poverty reduction intervention grounded in asset theory (Sherraden, 1991, 2016). Asset theory posits that accumulating assets enhances individuals’ and families’ well-being by providing a sense of security. Beyond improving financial status, assets contribute to a psychological sense of stability and future orientation, positively impacting family functioning (McKernan & Sherraden, 2008; Sherraden, 2016; Shobe & Page-Adams, 2001). In the context of family dynamics, asset accumulation can mitigate the negative impacts of financial strain by fostering an environment where families are less vulnerable to economic stressors. This reduction in vulnerability can lead to improved family functioning (Rothwell & Han, 2010).

Informed by asset theory, the intervention in this study aimed to mitigate the adverse effects of poverty by providing AIDS-orphaned children and their families with opportunities to accumulate assets through Child Development Accounts, structured as matched savings accounts. The primary goals of the intervention were to increase financial savings for the secondary education of participating children and adolescents and to offer support programs, including mentoring and educational activities, to reduce potential risks faced by AIDS-orphaned children. Children and adolescents participating in this study lived within family structures and were cared for either by a surviving parent (in the case of single orphans who had lost one parent to AIDS) or by extended family members, such as aunts, uncles, and grandparents (in the case of double orphans who had lost both parents to AIDS) (Nabunya et al., 2019).

The intervention was a five-year longitudinal cluster-randomized study conducted from 2011 to 2016 in Southern Uganda. Participants in the control group received “usual care” services typically provided to orphaned children and adolescents, including counseling (usually offered by church), school lunches, and scholastic materials such as textbooks, notebooks, and school uniforms. This was termed “usual care” because it included the standard support services provided to orphaned children and adolescents by local non-governmental organizations. The two treatment groups received all services included in the control condition, plus a bundled poverty reduction intervention comprising three components: (1) financial literacy training for participating children and their families provided by local partners; (2) a mentorship program following a nine-session curriculum to help participants set future goals and education aspirations; and (3) Child Development Accounts (CDA) held jointly in the name of the child and the caregiver at a formal financial institution (bank) registered with the Central Bank of Uganda. The study provided initial deposits, and savings accumulated in these accounts were matched by the program. The matching rate was the only difference between the two treatment arms: 1:1 in one treatment arm, and 2:1 in the other. The mentorship program, grounded in resilience theory (Fergus & Zimmerman, 2005), aimed to empower participating children and adolescents by guiding them to identify specific future goals and educational aspirations. The curriculum consisted of nine one-hour sessions, held monthly at the children’s schools over nine months. Topics included enhancing self-esteem, improving school attendance and grades, reducing stress, fostering hopefulness, developing stronger communication skills with caregivers and family members, promoting safe sexual decision-making, and reducing sexual risk-taking behavior. Mentors monitored attendance, addressed any missed sessions, and compiled mentoring reports after each session to document observations, challenges, and recommendations. Mentors were selected from local university students and project research assistant staff, following specific selection criteria and ensuring a gender match with the mentees (Ssewamala et al., 2014).

Previous studies have reported significant positive impacts of this intervention on crucial developmental and health outcomes among AIDS orphans. These impacts include improvements in children’s mental health (Karimli et al., 2023b; Karimli et al., 2019; Kivumbi et al., 2019), health functioning (Ssewamala et al., 2021), educational outcomes (Nabunya, 2019), and non-kin support networks for children (Nabunya et al., 2019). They also include reductions in child poverty (Wang et al., 2021) and increases in financial stability and material well-being (Wang et al., 2018). However, no study to date has examined the impact of this intervention on family functioning.

Empirical evidence from similar interventions suggests that asset accumulation can lead to positive changes in parental practices, such as increased involvement in children’s healthcare and education (Deng, 2019; Kim & Sherraden, 2011; Ssewamala & Ismayilova, 2009; Ssewamala et al., 2016). These interventions have also been found to alleviate parental stress (Huang et al., 2014; Nabunya et al., 2014; Wang et al., 2014), reduce punitive parenting practices (Huang et al., 2019), and improve family communication, fostering emotional connectedness, which benefits the development of AIDS-orphaned adolescents (Ismayilova et al., 2012). However, there is still a gap in research on the effects of asset accumulation interventions on various aspects of family functioning as reported by children themselves.

## Current Study

The current study focuses on the impact of a family-based poverty reduction intervention on family functioning, as reported by children. This emphasis on children’s perspectives is significant because it challenges traditional adult-centric approaches to family studies and provides unique insights into family dynamics from the children’s viewpoint. Understanding children’s perceptions and experiences is essential for grasping the impact of the family environment on their development and well-being. Our study contributes to the growing body of research aimed at understanding children’s lived experiences and how they perceive, interpret, and navigate their daily lives (Gross-Manos et al., 2021).

Furthermore, this study addresses a notable gap in the literature by examining potential variations in how these interventions affect family functioning based on a child’s gender and orphanhood status. This focus is crucial, as a child’s gender can significantly influence family dynamics, support systems, and responses to stressors. Extensive research highlights the importance of gender socialization and gender variations in child-parent relationships (Tenenbaum & May, 2013), emphasizing the need to address this gap. Evidence indicates significant gender differences in children’s relationships with caregivers and the unique roles that family support and specific caregivers play in children’s well-being (Colarossi & Eccles, 2003; Feldman et al., 2018; Figlio et al., 2019; Raley & Bianchi, 2006; Rueger et al., 2008; Van Polanen et al., 2017). Gender-based expectations and socialization practices within families often influence how children cope with stress and the support they receive. (Endendijk et al., 2016). Parental adherence to traditional gender roles shapes interactions and opportunities for children, impacting overall family dynamics (Few‐Demo & Allen, 2020). Studies suggest that communication patterns often reflect gender stereotypes, with a tendency to discuss emotions more with daughters than sons, affecting family bonds, support systems, and overall family functioning (Aznar & Tenenbaum, 2015; Chaplin et al., 2005; Fivush et al., 2019).

In contrast, the impact of orphanhood status on family functioning and the effectiveness of poverty reduction interventions is less understood, highlighting a significant gap in the literature. AIDS-orphaned children, in particular, face compounded challenges, such as the loss of primary caregivers, economic instability, and social stigma, which can exacerbate their vulnerabilities (Ashaba et al., 2019; Raymond & Zolnikov, 2018). These children often experience heightened economic stress and rely more heavily on extended family or community networks for support (Heymann et al., 2007; Karimli et al., 2012; Motha, 2018), where family functioning plays a crucial role in mitigating the effects of poverty and can lead to different dynamics and outcomes compared to children with living parents. Understanding how orphanhood status intersects with intervention outcomes is essential for tailoring effective support strategies and addressing the unique needs of these children.

Our study represents the first comprehensive investigation into the intervention’s effect on family functioning, with a specific focus on the nuanced effects of a child’s gender and orphanhood status. Drawing upon the asset theory framework discussed earlier, we also recognize the potential variations in how poverty and economic stressors influence family functioning relative to a child’s gender and orphanhood status. To address the existing research gap in this area, we investigate two primary research questions: (1) What is the effect of a family-based poverty reduction intervention on family functioning, as perceived and reported by children? (2) How does this impact differ based on the child’s gender and orphanhood status?

## Methods

### Study Design and Sampling

The study utilized data from 1410 children (n = 621 boys and n = 789 girls) enrolled in a three-arm randomized controlled trial. To prevent cross-arm contamination, randomization was conducted at the school level (n = 48). The initial screening covered 88 public primary schools across four study districts: Rakai, Masaka, Lwengo, and Kalungu in south-central Uganda. Among these, 48 schools were selected based on their medium-sized student population. Schools with enrollments exceeding 900 students were excluded, as they surpassed the average size, and those with fewer than 35 students per grade were also excluded, as they fell below the typical enrollment threshold of at least 50 students per grade. The final selection comprised schools that committed to participating in the study.

Recruitment relied heavily on collaboration with schools and local district administrations to identify eligible participants and facilitate enrollment. Schools organized informational meetings for families to learn about the study, during which research staff were present. These meetings provided parents with the opportunity to inquire about the study either individually or as part of a larger group. Children were eligible for enrollment if they met the following inclusion criteria: (1) being an AIDS orphan (having lost one or both parents to AIDS), (2) living in a family household, and (3) attending a public primary school at the fifth or sixth grade level (equivalent to sixth or seventh grades in the U.S. educational system). At the time of enrollment, the average age of children was 12.7 years, with ages ranging from 10 to 16 years.

Data were collected from the children and adolescents participating in the study over a period of five years (2011–2016) at the following intervals: baseline, 12 months, 24 months, 36 months, and 48 months. The intervention was administered over a 24-month period. By the end of the 48-month study period, the attrition rates were 8.8 and 10.6% for the two treatment arms, and 8.6% for the control arm. A design-based test for the independence of loss to follow-up indicated no significant differences in attrition rates between study conditions (Ssewamala et al., 2021). Additionally, we conducted Little’s MCAR test (Little, 1988) to determine if the missing data were independent of both observed and unobserved variables, indicating no systematic missingness. The test compares the means of each variable across different patterns of missing data and calculates a chi-square statistic based on the differences between observed and expected means under the MCAR assumption. Our results, with a p-value of 0.485, suggested that the data might be MCAR, as the probability of missingness appeared unrelated to the data itself. Given these results, we consider the data missing at random and apply complete case analyses to manage the missing data.

A 90-minute structured survey was administered by Ugandan interviewers who had been trained in good clinical practice and had obtained the Collaborative Institutional Training Initiative (CITI) Certificate before interacting with study participants. Informed assent was obtained from adolescents, and informed consent was obtained from their caregivers for participation in the study. The study protocol was approved by Columbia University Institutional Review Board (AAAI1950) and the Uganda National Council for Science and Technology (SS2586). The study protocol is registered at Clinicaltrial.gov (ID# NCT01447615).

### Measures

All measures used in this study had been previously tested in studies involving children and adolescents affected by AIDS in Uganda (Karimli et al., 2019; Ssewamala et al., 2009; Ssewamala et al., 2016; Ssewamala et al., 2021).

#### Outcome measures

Building on previous research that used the Family Assessment Measure (FAM) and Family Environment Scale (FES) to assess family dynamics (Booysen et al., 2021), we utilize the three measures detailed below to examine family functioning.

##### Family Cohesion

This measure, adapted from the Family Environment Scale (Moos & Moos, 1994) and the Family Assessment Measure (Skinner et al., 2009), was assessed using a composite score of six items. These items captured aspects such as whether family members sought help from each other before seeking assistance from non-family members, whether they enjoyed spending leisure time together, feelings of closeness among family members, availability for communication, and shared activities as a family. Each item was rated on a 5-point scale (0 = Never to 4 = Always). The composite score ranged from 0 to 24, with higher scores indicating greater levels of family cohesion. The scale demonstrated high internal reliability, with Cronbach’s alpha ranging from 0.72 at baseline to 0.77 at Wave 5

##### Family Communication

This measure, adapted from the Family Environment Scale (Moos & Moos, 1994) and the Family Assessment Measure (Skinner et al., 2009), consisted of two separate sub-scales:

##### Frequency of Family Communication

This sub-scale was a composite score of twelve items reflecting the frequency with which children and adolescents communicated with their caregivers on specific topics, such as education, future planning, risk-taking behaviors, and HIV/AIDS. Each item was rated on a 5-point scale (0 = Never to 4 = Always). The composite score ranged from 0 to 48, with higher scores indicating a higher frequency of communication between the child and caregiver. The scale demonstrated high internal reliability, with Cronbach’s alpha ranging from 0.81 at baseline to 0.83 at Wave 5.

##### Comfort of Family Communication

This sub-scale was a composite score of twelve items reflecting the level of comfort children and adolescents felt when communicating with their caregivers about topics such as education, future planning, risk-taking behaviors, and HIV/AIDS. Each item was measured on a 5-point scale (0 = Very Uncomfortable to 4 = Very Comfortable). The composite score ranged from 0 to 48, with higher scores indicating a greater level of comfort in communication. Similar to the frequency sub-scale, this scale also demonstrated high internal reliability, with Cronbach’s alpha ranging from 0.85 at baseline to 0.87 at Wave 5.

##### Child-Caregiver Relationship

This measure, adapted from the Social Support Behavior Scale (SS-B) (Vaux et al., 1987), was developed through factor analysis. The Kaiser-Meyer-Olkin test for sampling adequacy yielded a value of 0.88, exceeding the recommended threshold of 0.6 (Watkins, 2021). A total of twenty-six items from the survey instrument were included in the factor analysis. Both scree plot analyses and Parallel Analysis for Principal Components suggested a three-factor structure. Varimax rotation revealed significant loading of variables onto two distinct factors, indicating an inherent structure (Pett et al., 2003). Factor one accounted for 77% of the variance, while factor two accounted for 24%. Out of the initial 26 items, eleven demonstrated strong factor loadings (>0.3) on one factor with an eigenvalue of 3.94. The remaining variables, although loading onto another factor (with an eigenvalue of 1.23), formed a scale with Cronbach’s alpha below the recommended level (<0.6), indicating inadequate internal reliability. Consequently, these items were excluded from further analysis. The final scale comprised eleven questions assessing the quality of relationships between children and the adults they lived with. Responses were rated on a 5-point Likert scale (0 = Never to 4 = Always). The questions addressed various aspects, including whether the child could rely on the caregiver for assistance, whether they felt encouraged by the caregiver to excel, whether the caregiver provided explanations for tasks, familiarity with the child’s friends, conversational engagement, shared activities, attentive listening, emotional support, recognition of the child’s importance, and acceptance of the child’s individuality. The total score ranged from 0 to 44, with higher scores indicating a higher quality of the child-caregiver relationship. The scale demonstrated high internal reliability, with Cronbach’s alpha ranging from 0.75 at baseline to 0.84 at Wave 5.

#### Intervention

As previously described, the intervention included two treatment arms, with the match rate (i.e., financial incentive for saving) being the only difference between them. The average amount of savings accumulated in this intervention was around US$ 25 over the intervention period (Wang et al., 2021), which does not hold conceptual significance for the purposes of our study. Therefore, our analyses combined both arms into a single treatment group, creating a binary measure of intervention (0=control group; 1=treatment group).

### Statistical Analyses Procedures

We followed the CONSORT (Appendix 1) guidelines to report baseline sample characteristics. To examine baseline differences across the study arms, we reported adjusted Wald F-statistics (design-based F), which account for individual-level variations and potential between-school correlations.

The analyses, run in Stata 16, accounted for the multilevel nature of data with repeated measures being nested within individuals, time points, and schools. Given the randomization at the school level, there was a potential risk of correlation for within-school observations, which could violate the independence assumption (Raudenbush & Bryk, 2002). To address this, we ran multilevel mixed-effects models, incorporating both between-school and within-individual variability as random effects, to estimate subject-specific effects while accounting for school-level clustering and potential within-individual correlations (Gelman & Hill, 2006). Finally, we decomposed the effects into comparisons against reference categories to obtain the marginal treatment effect at each time point.

To answer our first question (What is the effect of a family-based poverty reduction intervention on family functioning, as perceived and reported by children?) we ran multilevel mixed-effects models as specified below:

(1) $${\text{Y}}_{\text{it}}=\alpha_{0}+\beta_{1}{\text{I}}_{\text{i}}+\beta_{2}{\text{T}}_{\text{it}}+\beta_{3}\left({\text{I}}_{\text{i}}*{\text{T}}_{\text{it}}\right)+{\text{u}}_{\text{s}}+{\text{e}}_{\text{t}}+{\text{z}}_{\text{i}}$$

Here, ${\text{Y}}_{\text{it}}$ was the continuous outcome for the i-th observations $\left(\text{i}=1,2,\ldots 1410\right)$ at time $\text{t}\left(\text{t}=1,2\ldots 5\right)$; I was treatment (I=0 for control group; D=1 for treatment group); T was time (T=1 at baseline; T=2 at 12 months; T=3 at 24 months; T=4 at 36 months; and T=5 at 48 months); ${\text{u}}_{\text{s}}$ was the level 1 error (i.e. differences between the expected and observed values of outcome at school level); ${\text{e}}_{\text{t}}$ was the level 2 error (i.e. difference between the expected and observed values of outcome at time level); and ${\text{z}}_{\text{i}}$ was the level 3 error (i.e. difference between the expected and observed values of outcome at individual level). Robust standard errors were adjusted for clustering within schools to estimate subject-specific effects accounting for school-level clustering (Hox et al., 2017). Treatment effects were reported as time-within-group simple effect comparisons (i.e., treatment arm vs. control arm at each time point) obtained via multiple pairwise comparison analyses. We used Sidak’s adjustment method (Abdi, 2007), that mitigates the false discovery risk by offering p-value corrections for multiple comparisons.

To answer our second question (Does the effect of a family-based poverty reduction intervention on family functioning vary by child’s gender and orphanhood status?), we conducted two separate moderator analyses. These analyses examined whether the child’s gender and orphanhood status moderate (i.e., shape the direction and/or strength of) the intervention’s effect on family functioning. To assess these potential moderation effects, we ran two regression models, each incorporating a different three-way interaction term to the model described in Eq. (1): the group-by-time-by-child’s gender interaction and the group-by-time-by-child’s orphanhood status interaction. This approach allowed us to independently assess the moderating effects of child’s gender and orphanhood status. We assessed the moderation effects through joint tests of these three-way interactions, which demonstrate the interaction between time and group on the slopes of gender and orphanhood status. Specifically, these tests assessed whether the intervention’s effect over time differed by gender (boys versus girls) and across different orphanhood categories. The orphanhood categories were double orphans (children who have lost both parents to AIDS), single paternal orphans (children who have lost their fathers to AIDS but have a living mother), and single maternal orphans (children who have lost their mothers to AIDS but have a living father). To further explore these three-way interactions and understand the intervention’s effect on family functioning within each group, we conducted simple slope analyses using the “margins” command in Stata 16. This method allowed us to examine the effect of the intervention within each subgroup defined by gender and orphanhood category without cross-group comparisons. In these simple slope analyses, at each time point, participants in the control group within the same subgroup served as the reference group for comparison. For example, within the “single paternal orphans” category, we compared participants in the treatment arm to those in the control arm at each time point, without comparing them to participants in other orphanhood categories. This approach provided a clearer understanding of how the intervention affected family functioning within each subgroup, highlighting the nuances of its impact based on gender and orphanhood status.

## Results

Table 1 describes baseline characteristics of our sample.

At baseline, the majority of children were identified as single maternal orphans, while approximately one-fifth were single paternal orphans, and another one-fifth were double orphans.

Results (Table 2) show significant positive effect of the intervention on family communication, including both the frequency of communication and the comfort of communication, in the short term.

More specifically, at 12 months, participants in the treatment group reported a higher frequency of communication and greater comfort in communicating with their caregivers compared to their control group counterparts. The significant positive effect of the intervention on communication frequency persisted at the 24-month follow-up but eventually faded away. The results indicate no significant effect of the intervention on either family cohesion or the child-caregiver relationship.

Moderator analyses and joint three-way interaction tests (Table 3) indicate that the intervention’s effect on family functioning was not significantly moderated by the child’s gender. However, simple slope analyses (Table 3) show a significant positive effect of the intervention on family communication, including both the frequency of communication and comfort in communicating, for boys in the short term. In particular, compared to their counterparts in the control group, boys in the treatment group reported higher frequency of communication at the 12-month and 24-month follow ups. They also reported greater comfort in communicating, but only at the 12-month follow-up. The seeming inconsistency between these findings can be attributed to the distinct methodological approaches of the two types of analyses. Moderator analyses and three-way interaction tests are designed to evaluate whether the effect of the intervention varies across distinct levels of the moderator variable—here, the child’s gender—across the entire sample. Such tests may not yield statistically significant results if the overall moderating effect is weak or displays variability across time points. Conversely, simple slope analyses are focused on examining specific subgroups and discrete time points, which allows for the detection of significant effects within these more narrowly defined contexts. This approach can reveal a positive intervention effect on communication for boys at particular follow-up intervals, even in cases where the overall moderation effect is not statistically significant.

Girls, on the other hand, experienced significant positive effects of the intervention on family cohesion and the child-caregiver relationship. More specifically, at the 24-month follow-up, girls in the treatment group reported greater family cohesion and better child-caregiver relationship than girls in the control group.

Moderator analyses and three-way interaction tests (Table 4) show that a child’s orphanhood status moderates only the intervention’s effect on the child-caregiver relationship. Simple slope analyses (Table 3) show a significant and lasting positive effect (at 24, 36, and 48 months) of the intervention on this outcome for paternal orphans only. We found no significant intervention effect on this outcome for other orphanhood categories. For single maternal orphans, the results show a significant positive effect of the intervention on family communication, including both the frequency of communication and comfort of communicating. Compared to their counterparts in the control group, single maternal orphans in the treatment group reported a higher frequency of communication at the 12-month and 24-month follow ups. They also reported greater comfort in communicating at 12, 24, 36, and 48 months following the baseline assessment.

For double orphans, the results show no positive effect of the intervention on any of the family functioning outcomes. On the contrary, the results suggest that at 36 months, double orphans in the treatment group reported lower levels of communication comfort than their counterparts in the control group.

## Discussion

Our research is among the few studies in the field that employ an experimental design to rigorously evaluate the effects of an asset-based poverty reduction intervention on family functioning. This contribution is significant, given the well-documented role of family functioning as a protective factor against adverse experiences and its importance as a predictor of positive health outcomes for vulnerable children and adolescents worldwide (WHO, 2016). A robust evaluation of poverty reduction interventions’ impact on family functioning is crucial, as these interventions have substantial potential to mitigate the long-term effects of poverty—such as economic stress and family dysfunction—on adverse childhood experiences and subsequent physical and mental health outcomes in adulthood (Chen et al., 2017; Choi et al., 2019).

Our findings present a nuanced perspective that partially aligns with the hypotheses proposed by asset theory (McKernan & Sherraden, 2008; Sherraden, 1991, 2016). We found significant intervention effects on family communication, including the frequency and comfort of communication. However, these effects were not sustained beyond the 24-month intervention period. The mentorship component, which included sessions focused on developing stronger communication skills with caregivers and family members, likely contributed to the observed improvements in family communication during the 24-month intervention period. Once the intervention was concluded, its significant positive effects on family communication dissipated. Furthermore, on average, we found no significant intervention effects on family cohesion or the child-caregiver relationship.

Poverty reduction interventions that enhance a family’s financial resources address only one of the many protective factors within a family system that contribute to the family’s resilience (Henry et al., 2015). Other aspects of family functioning, such as family cohesion and the quality of child-caregiver relationships, might be more deeply rooted in the emotional and psychological structure of the family, requiring additional resilience-promoting interventions (Black & Lobo, 2008). Family strengthening interventions aimed at improving intra-family relationships and fostering a supportive family environment can effectively complement economic support initiatives. Recent evidence (Karimli et al., 2023a) suggests that combining asset accumulation interventions with multifamily group (MFG) therapy, which focuses on therapeutic processes like child management techniques and emotional regulation, significantly enhances family cohesion and the quality of child-caregiver relationships. This aligns with earlier research (Ismayilova & Karimli, 2020) indicating that combination of poverty reduction intervention with family coaching centered around family communication on child protection issues can substantially improve child-caregiver relationships and parenting practices in low-income settings. These examples emphasize the importance of an integrated, comprehensive approach that combines economic assistance with psychosocial and behavioral interventions to address the multifaceted nature of family dynamics. Further research is essential to explore and refine integrated models for supporting low-income families and to establish a comprehensive framework for enhancing family functioning among economically disadvantaged families.

We found no evidence that the child’s gender moderated the effect of the intervention on family functioning. In contrast, the child’s orphanhood status significantly moderated the effect of the intervention on the child-caregiver relationship. Additional simple slope analyses, conducted for each category of the moderator variables (i.e., child’s gender and orphanhood status), revealed significant variations in intervention effects, suggesting potential nuances within specific subpopulations. It is important to interpret these subgroup differences cautiously and avoid overstating their significance, especially given the absence of a significant overall moderation effect. However, the findings are intriguing and offer valuable insights for further exploration. The emergence of significant results within certain subgroups, despite a non-significant three-way test of moderation, may be due to the increased sensitivity of simple slope analyses in detecting variations within specific categories. The three-way interaction test evaluates whether the relationship between the intervention and outcomes differs consistently across all combinations of the moderator variables. A non-significant result in this test does not rule out the possibility of significant effects within individual subgroups. Subgroup analyses can uncover differential intervention effects that are contextually dependent and may not be evident in broader interaction tests. Consequently, these findings, although preliminary, provide valuable insights into the complex interplay between intervention effects and specific characteristics, warranting further investigation into these nuanced dynamics.

More specifically, we found improved family communication (including the frequency and comfort of communication) for boys, and improved family cohesion and quality of the child-caregiver relationship for girls. Similarly, single maternal orphans (i.e., children who lost their mothers) in the treatment groups reported improved family communication, whereas single paternal orphans (i.e., children who lost their fathers) in the treatment group reported improved child-caregiver relationship. This finding aligns with previous studies that suggest significant gender variations in family functioning outcomes and processes (Colarossi & Eccles, 2003; Feldman et al., 2018; Figlio et al., 2019; Raley & Bianchi, 2006; Rueger et al., 2008; Van Polanen et al., 2017). Our results can be contextualized within the framework of gender schema theory (Bem, 1983; Starr & Zurbriggen, 2017), gender socialization theory (Leaper & Friedman, 2007), and the hypothesis that children develop closer relationships with the same-gender caregivers(Van Polanen et al., 2017). Previous research (Karimli et al., 2012) has highlighted the predominant role of female caregivers for children in our sample. Consequently, considering the proposition that girls might develop more secure attachment relationships with female caregivers and boys with male caregivers (Van Polanen et al., 2017), it is possible that the intervention specifically improved the quality of relationships with primarily female caregivers and fostered a greater sense of family cohesion among girls, but not among boys. Moreover, gender-based socialization patterns can contribute to variations in how children perceive and respond to interventions, as well as the support they receive from their families (Endendijk et al., 2016). In Uganda, traditional gender norms are deeply rooted in societal expectations and cultural practices. Women are often expected to fulfill nurturing roles within the household, focusing on caregiving and maintaining family cohesion, while men are typically associated with providing and less involved in emotional caregiving (Ninsiima et al., 2018). This division of gender roles can influence how children are socialized from a young age, reinforcing the idea that emotional expression and communication are more acceptable for girls than boys (Nalukwago et al., 2019; Vu et al., 2017). These traditional gender norms and practices extend to parental communication styles with their children, as parents and caregivers often reinforce traditional gender stereotypes by showing a greater inclination to discuss emotions with daughters compared to sons (Aznar & Tenenbaum, 2015; Chaplin et al., 2005; Fivush et al., 2019). These gender-related dynamics may help understand the observed intervention effects, which showed improvement in family communication for boys, and in child-caregiver relationships and family cohesion for girls.

### Limitations

This study has two key limitations to be considered when interpreting the findings. First, the absence of caregiver data limits our insight into family functioning, which is based solely on children’s accounts of family cohesion, the child-caregiver relationship, and communication within the family. The lack of caregiver-reported data, particularly regarding parenting stress, restricts our understanding of the caregiver’s perspective on these relationships. Furthermore, we have no data from various family members to assess the strengths and challenges of the family, as well as the communication patterns and capabilities that define the household’s dynamics. Without these broader measures and viewpoints, the study falls short of capturing the full complexity of the family environment.

Second, the study does not capture the nuances of intra-household dynamics, particularly the complexity of care arrangements for orphaned children. These children may experience various care structures offered by their extended families (Bryant & Beard, 2016; Heymann et al., 2007; Karimli et al., 2012). For instance, a maternal orphan—a child who lost their mother—might reside with their father but depend primarily on their grandmother for daily sustenance and emotional support. Our dataset does not specify the caregivers’ roles and contributions, leaving a gap in our comprehension of who provides critical forms of care such as emotional support, supervision, and basic needs. Without this detailed understanding, we cannot fully appreciate the intricate fabric of family functioning in these households, nor can we properly acknowledge the contributions of extended family members who may play pivotal roles in the lives of these children. Despite its limitations, our study is an important contribution to the robust examination of effects of poverty reduction interventions on family functioning, providing valuable insights from children’s perspectives.

### Implications

Assessing the impact of poverty reduction interventions on family functioning is important, with economic strengthening serving as a means to the broader objective of emotional and social well-being of a family. When asset accumulation and poverty reduction alone is insufficient, additional resources need be invested in complementary programs to ensure more lasting and robust effects on family functioning. Our findings indicate that asset accumulation and economic strengthening alone may not substantially improve family functioning, emphasizing the need for a comprehensive, multifaceted approach to fostering family resilience. This may contribute to a shift in policy and intervention design towards an integrated approach, addressing both economic and psychosocial indicators of family functioning and family well-being.

Further research is needed to identify resilience-strengthening interventions that effectively complement poverty reduction and asset accumulation initiatives, while considering the diverse cultural and socioeconomic backgrounds of populations. Gathering comprehensive data from family members to fully capture the complexity of family functioning will enhance this field.

## Conclusions

In summary, our findings indicate that the asset-based poverty reduction intervention targeting poor families in Southern Uganda, specifically those caring for AIDS orphaned children, has a limited effect on family functioning. Our study suggests that poverty reduction interventions may need to be combined with other resilience-strengthening measures to achieve lasting and robust improvements in family functioning, especially among families caring for AIDS orphaned children in a low-income setting. Furthermore, our study suggests that the effects of these interventions on specific aspects of family functioning may vary depending on the gender and orphanhood status of the children. These variations have not been reported in previous studies, emphasizing the need for continued further research in this underexplored area. Our findings also highlight a need for comprehensive consideration of complexities related to interpersonal aspects, gender variations, and family structures when designing poverty reduction interventions, aiming for their impact to extend beyond mere material well-being.

## Figures and Tables

**Table 1:** Baseline characteristics of the study sample

Variables	Control group (*n* = 496)		Treatment group (*n* = 914)		Total (*n* = 1410)		Design-based F
	Percentage or mean [95%] confidence intervals						
Family cohesion (range: 0–24)	17.8	[16.9; 18.7]	17.3	[16.8; 17.9]	17.5	[17; 18]	0.83
Frequency of family communication (0–48)	15.8	[15; 16.5]	15.9	[15.1; 16.8]	15.9	[15.3; 26.5]	0.08
Comfort of family communication (0–48)	11.6	[10.9; 12.2]	11.3	[10.8; 11.8]	11.4	[11; 11.8]	0.32
Child-caregiver relationship (Range: 0–44)	32.7	[31.8; 33.6]	32.1	[31.5; 32.7]	32.3	[31.8; 32.8]	1.27
Child’s gender							0.63
Male	44.96	[40.4; 49.6]	43.54	[39.9; 47.2]	44.04	[41.2; 46.9]	
Female	55.04	[50.4; 59.6]	56.46	52.8; 60.1]	55.96	[53.1; 58.8]	
Child’s orphanhood status							2.24
Double orphan	22.8	[19.9; 26]	17.8	[14.9; 21.2]	19.6	[17.3; 22.1]	
Single paternal orphan	22.8	[17.3; 29.3]	21.3	[18.5; 24.5]	21.8	[19.1; 24.9]	
Single maternal orphan	54.4	[49.2; 59.6]	60.8	[57.5; 64.1]	58.6	[55.6; 61.5]	

**Table 2:** Effect of the poverty reduction intervention on family functioning, over the course of 48 months

Variables	Family cohesion		Frequency of communication		Comfort of communication		Child-caregiver relationship	
	EMD^a^	95% CI	EMD^a^	95% CI	EMD^a^	95% CI	EMD^a^	95% CI
**Treatment Arm vs. Control Arm**								
at 12 months	0.26	[−0.5; 1]	1.58*	[0.4; 2.8]	1.07*	[0.2; 1.9]	0.18	[−0.8; 1.1]
at 24 months	0.62	[−0.2; 1.4]	1.71*	[0.3; 3.1]	0.89	[−0.3; 2.1]	0.95	[−0.2; 2.1]
at 36 months	0.37	[−0.5; 1.2]	0.42	[−1.1; 1.9]	0.17	[−0.8; 1.1]	0.53	[−0.5; 1.6]
at 48 months	0.02	[−0.6; 0.6]	0.37	[−1; 1.8]	0.62	[−0.2; 1.4]	0.39	[−0.4; 1.2]
Observations	6,429		6,408		6,407		6,425	
Number of groups	48		48		48		48	

*** *p* < 0.001

** *p* < 0.01

* *p* < 0.05

a EMD differences of estimated marginal means between treatment and control group at each time point. These coefficients represent group-within-time simple effects

**Table 3:** Effect of the poverty reduction intervention on family functioning by child’s gender

Variables	Family cohesion		Frequency of communication		Comfort of communication		Child-caregiver relationship	
Joint test of three-way interaction effect	χ^2^ (4) = 2.82; *P* = 0.59		χ^2^ (4) = 6.56; *P* = 0.16		χ^2^ (4) = 3.15; *P* = 0.53		χ^2^ (4) = 6.06; *P* = 0.19	
	EMD^(a)^	95% CI	EMD^(a)^	95% CI	EMD^(a)^	95% CI	EMD^(a)^	95% CI
**Treatment Arm vs. Control Arm for Boys**								
at 12 months	0.16	[−0.8; 1.1]	1.52*	[0.2; 2.9]	1.13*	[0.1; 2.2]	−0.02	[−1.5; 1.4]
at 24 months	−0.02	[−1.1; 1]	1.81*	[0.3; 3.3]	0.80	[−0.8; 2.4]	−0.13	[−1.7; 1.5]
at 36 months	0.27	[−0.8; 1.3]	0.66	[−1.3; 2.6]	0.07	[−1.5; 1.6]	0.49	[−1; 1.9]
at 48 months	−0.08	[−0.8; 0.7]	−0.43	[−2.4; 1.5]	1.31	[−0.4; 3]	0.51	[−0.9; 1.9]
Observations	2,905		2,892		2,891		2,903	
Number of groups	48		48		48		48	
**Treatment Arm vs. Control Arm for Girls**								
at 12 months	0.35	[−0.6; 1.3]	1.51	[−0.2; 3.2]	0.96	[−0.2; 2.1]	0.41	[−0.9; 1.7]
at 24 months	1.17*	[0.2; 2.1]	1.50	[−0.4; 3.4]	0.87	[−0.3; 2.1]	1.92*	[0.4; 3.4]
at 36 months	0.48	[−0.7; 1.6]	0.13	[−1.6; 1.8]	0.19	[−0.9; 1.3]	0.63	[−0.7; 2]
at 48 months	0.14	[−0.7; 1]	1.00	[−0.8; 2.8]	0.01	[−0.8; 0.8]	0.36	[−0.9; 1.6]
Observations	3,524		3,516		3,516		3,522	
Number of groups	48		48		48		48	

*** *p* < 0.001

** *p* < 0.01

* *p* < 0.05

a EMD is differences of estimated marginal means. These coefficients represent group-within-time simple effects

**Table 4:** Effect of the poverty reduction intervention on family functioning by child’s orphanhood status

VARIABLES	Family cohesion		Frequency of communication		Comfort of communication		Child-caregiver relationship	
Joint test of three-way interaction effect	χ^2^ (8) = 12.45; *P* = 0.13		χ^2^ (8) = 7.9; *P* = 0.44		χ^2^ (8) = 9.96; *P* = 0.27		χ^2^ (8) = 15.93*; *P* = 0.04	
	EMD^a^	95% CI	EMD^a^	95% CI	EMD^a^	95% CI	EMD^a^	95% CI
**Treatment Arm vs. Control Arm for Double Orphans**								
at 12 months	−0.36	[−1.4; 0.7]	1.47	[−0.5; 3.4]	0.04	[−1.5; 1.6]	−1.49	[−3.5; 0.5]
at 24 months	0.38	[−1.1; 1.9]	0.96	[−1.5; 3.4]	−0.41	[−2.7; 1.9]	−0.30	[−2.6; 2.0]
at 36 months	−0.19	[−1.8; 1.5]	−1.04	[−3.4; 1.3]	−1.93*	[−3.7; −0.2]	−0.83	[−3.1; 1.4]
at 48 months	0.46	[−0.9; 1.8]	0.44	[−1.6; 2.5]	−0.50	[−2.7; 1.7]	−0.20	[−2.6; 2.2]
Observations	1,248		1,243		1,243		1,247	
Number of groups	47		47		47		47	
**Treatment Arm vs. Control Arm for Single Paternal Orphans**								
at 12 months	−0.35	[−1.6; 0.9]	−0.06	[−3; 2.9]	0.48	[−1.1; 2.1]	0.81	[−1.4; 3]
at 24 months	0.98	[−0.5; 2.5]	1.31	[−1; 3.6]	0.12	[−1.7; 1.9]	2.6*	[0.5; 4.7]
at 36 months	1.34	[−0.2; 2.9]	0.82	[−1.2; 2.8]	−0.83	[−2.9; 1.2]	2.54*	[0.5; 4.5]
at 48 months	0.44	[−0.9; 1.7]	−0.05	[−2.9; 2.8]	0.33	[−1.3; 2.0]	2.69*	[0.6; 4.8]
Observations	1,397		1,394		1,393		1,395	
Number of groups	48		48		48		48	
**Treatment Arm vs. Control Arm for Single Maternal Orphans**								
at 12 months	0.73	[−0.2; 1.7]	2.13**	[0.7; 3.6]	1.61**	[0.4; 2.8]	0.35	[−0.8; 1.5]
at 24 months	0.48	[−0.6; 1.6]	2.09*	[0.03; 4.2]	1.74**	[0.5; 2.9]	0.59	[−1.0; 2.1]
at 36 months	0.14	[−0.8; 1.1]	0.71	[−1.2; 2.6]	1.39**	[0.4; 2.4]	0.09	[−1.1; 1.2]
at 48 months	−0.29	[−1.1; 0.5]	0.46	[−1.4; 2.3]	1.22*	[0.2; 2.3]	−0.54	[−1.7; 0.6]
Observations	3,784		3,771		3,771		3,783	
Number of groups	48		48		48		48	

*** *p* < 0.001

** *p* < 0.01

* *p* < 0.05

a EMD is differences of estimated marginal means. These coefficients represent group-within-time simple effects

**Fig. 1:**
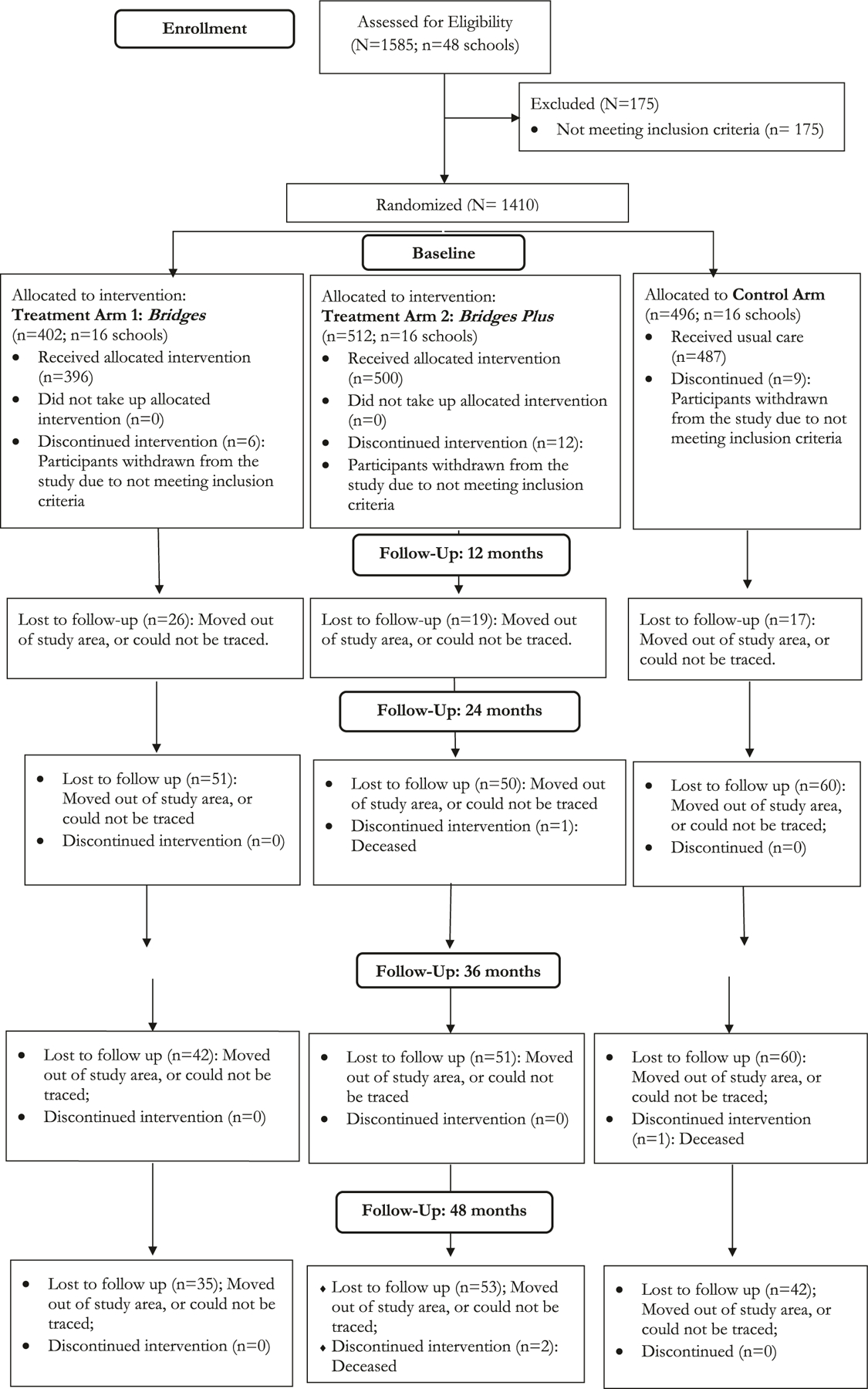
Consort Chart

## Highlights

- Poverty reduction interventions are important to improve family functioning for low-income families in Sub-Saharan Africa.
- Asset-based poverty-reduction intervention improves family functioning among families caring for AIDS orphans in Uganda.
- Effects vary by child’s gender and orphanhood category, which has not been reported in previous studies.
- Findings suggest a need for comprehensive consideration of complexities related to gender variations and family structures.
